# A motor imagery classification model based on hybrid brain-computer interface and multitask learning of electroencephalographic and electromyographic deep features

**DOI:** 10.3389/fphys.2024.1487809

**Published:** 2024-12-05

**Authors:** Yingyu Cao, Shaowei Gao, Huixian Yu, Zhenxi Zhao, Dawei Zang, Chun Wang

**Affiliations:** ^1^ College of Mechanical Engineering, Beijing Institute of Petrochemical Technology, Beijing, China; ^2^ Department of Rehabilitation, Beijing Tian Tan Hospital, Capital Medical University, Beijing, China; ^3^ College of Mechanical Engineering, Tiangong University, Tianjin, China

**Keywords:** motor imagery, hybrid brain-computer interface, variational autoencoder, channel attention mechanism, multitask learning

## Abstract

**Objective:**

Extracting deep features from participants’ bioelectric signals and constructing models are key research directions in motor imagery (MI) classification tasks. In this study, we constructed a multimodal multitask hybrid brain-computer interface net (2M-hBCINet) based on deep features of electroencephalogram (EEG) and electromyography (EMG) to effectively accomplish motor imagery classification tasks.

**Methods:**

The model first used a variational autoencoder (VAE) network for unsupervised learning of EEG and EMG signals to extract their deep features, and subsequently applied the channel attention mechanism (CAM) to select these deep features and highlight the advantageous features and minimize the disadvantageous ones. Moreover, in this study, multitask learning (MTL) was applied to train the 2M-hBCINet model, incorporating the primary task that is the MI classification task, and auxiliary tasks including EEG reconstruction task, EMG reconstruction task, and a feature metric learning task, each with distinct loss functions to enhance the performance of each task. Finally, we designed module ablation experiments, multitask learning comparison experiments, multi-frequency band comparison experiments, and muscle fatigue experiments. Using leave-one-out cross-validation(LOOCV), the accuracy and effectiveness of each module of the 2M-hBCINet model were validated using the self-made MI-EEMG dataset and the public datasets WAY-EEG-GAL and ESEMIT.

**Results:**

The results indicated that compared to comparative models, the 2M-hBCINet model demonstrated good performance and achieved the best results across different frequency bands and under muscle fatigue conditions.

**Conclusion:**

The 2M-hBCINet model constructed based on EMG and EEG data innovatively in this study demonstrated excellent performance and strong generalization in the MI classification task. As an excellent end-to-end model, 2M-hBCINet can be generalized to be used in EEG-related fields such as anomaly detection and emotion analysis.

## 1 Introduction

Movement is a fundamental physiological activity in humans that is essential for maintaining daily life and work. Currently, human movement intention detection has significant potential in the field of artificial intelligence. The brain-computer interface (BCI) (Vidal, 1973) technology used to recognize human movement intentions is referred to as motor imagery (MI) task. MI has widespread applications in rehabilitation therapy (Li et al., 2014), autonomous driving (Yan et al., 2024), and entertainment (Kilteni et al., 2018), making it a research hotspot in the BCI field. The mainstream method for performing MI tasks involves analyzing electroencephalogram (EEG) signals to identify brainwave patterns corresponding to motor intentions, thereby recognizing human motor intentions.

The current methods used by researchers to improve the accuracy of MI tasks can be categorized into the following two types: 1. Continuous optimization and improvement of signal acquisition and classification models (Sreeja et al., 2017; Ravindran and Vinod, 2019), 2. Expanding the scope of information analysis by integrating multiple types of information to achieve the MI task, a method known as hybrid BCI (hBCI) technology. The hBCI has evolved from traditional BCI and offers advantages such as strong stability and high accuracy (Zhang et al., 2007). Depending on their construction, hBCI systems can be divided into three categories: 1. Systems based on the fusion of various EEG paradigms, such as the combination of steady-state visual evoked potentials (SSVEP) and MI paradigms, which enable precise control of complex devices such as robotic arms (Duan et al., 2015); 2. hBCI systems constructed using multimodal stimulation methods, which include the collaborative regulation of SSVEP amplitude (Li et al., 2019) through visual and tactile stimuli to enhance system response, as well as the integration and optimization of visual and auditory signals for the rehabilitation of patients with consciousness disorders, significantly enhancing rehabilitation efficiency (Pan et al., 2018). 3. hBCI systems based on the fusion of various physiological signals, such as the combined use of EEG and electrooculogram (EOG) signals, to significantly boost target recognition accuracy, particularly in target selection tasks, and the deep integration of EEG and electromyography (EMG) signals to further enhance the performance of MI classification tasks (Hooda et al., 2020). Because motor control is directly related to muscle activity, this area is considered a research hotspot and frontier in hBCI.

In the field of bioelectric signal processing, various methods have been proposed for effectively analyzing data and completing MI classification tasks. Traditional machine learning methods primarily extract time-domain, frequency-domain, time-frequency, and nonlinear features from EEG and EMG signals (Jenke et al., 2014; Li et al., 2018), and classify these features using algorithms such as support vector machines (SVM) and k-nearest neighbors (KNN) (Mohammadi et al., 2017; George et al., 2019). However, traditional machine learning methods have limited generalizability, require relevant domain knowledge and expert experience, and cannot completely extract and elucidate the deep features of EEG and EMG signals. With continuing advances in deep learning technology, an increasing number of deep learning models that possess end-to-end capabilities and enhanced performance are being applied to MI classification tasks.

In summary, although current methods for MI classification have achieved significant results, they still have certain limitations. These include heavy reliance on single signal sources such as EEG or EMG, which restricts the comprehensiveness of information and makes the system susceptible to interference, thereby reducing classification accuracy and robustness. EEG captures electrical signals generated by brain activity; however, it is highly susceptible to noise and has significant individual variability. Although EMG signals can directly reflect muscle activity, prolonged or high-intensity usage can lead to muscle fatigue, significantly affecting the stability and reliability of EMG signals, and complicating continuous and effective MI classification. Traditional machine learning methods rely on expert knowledge for feature extraction, which limits their ability to generalize and automate, making it challenging to achieve optimal classification performance. Supervised learning can only extract specific features that are typically selected based on labeled data. However, in real-world applications, the label information may not always be available or accurate. Additionally, although some deep learning models possess strong feature extraction capabilities, their performance may be compromised if multimodal information is not completely utilized. These models also face the challenge of balancing complexity and generalization capability.

To address these issues, we propose an end-to-end hBCI model known as 2M-hBCINet, which significantly enhances the accuracy and robustness of classification tasks by fusing EEG and EMG signals. Compared to methods that depend solely on EEG or EMG as a single signal source, this model can comprehensively capture complementary information between brain and muscles during the expression of motor intentions. The model utilized a variational autoencoder (VAE) to extract deep features from EEG and EMG signals and then employed a channel attention mechanism (CAM) to assign varying weights to these features, thereby achieving the goal of feature selection. Subsequently, multitask learning (MTL) was adopted, with MI classification task as the primary task and EEG reconstruction, EMG reconstruction, and feature metric learning as the auxiliary tasks. The concurrent training of these tasks enhanced the model’s performance and generalizability. Additionally, we conducted module ablation experiments to verify the effectiveness of each component within the 2M-hBCINet model, multitask ablation experiments to confirm the efficacy of multitask training, and validated the model’s superiority under various frequency bands (theta (four to eight Hz), alpha (8–13 Hz), beta (13–30 Hz), and gamma (30–50 Hz), as well as under different levels of muscle fatigue. The main contributions of this study are as follows.

1) We propose an MI classification network model called 2M-hBCINet, based on EEG and EMG data. Unsupervised methods were used to extract deep features of EEG and EMG through VAE networks, the features were combined, and CAM was then used to select features.
2) We used an MTL approach to train the model. The primary task in MTL is the MI classification task, and auxiliary tasks include EEG reconstruction, EMG reconstruction, and feature metric learning tasks. This effectively improved the accuracy of the model and enhanced its generalizability.
3) We constructed a self-made dataset called MI-EEMG, collecting EEG and EMG data from 14 participants during MI tasks.
4) Through experiments, we verified the effectiveness of the proposed 2M-hBCINet model. We performed extensive experimental validation using the leave-one-out cross-validation (LOOCV) method, with validation datasets including the self-made dataset MI-EEMG and public datasets WAY-EEG-GAL (Luciw et al., 2014), and Electrophysiological Signals of Embodiment and Ml-BCI Training in virtual reality (VR) (referred to as the ESEMIT dataset in the following text) (Katarina and Athanasios, 2023). The experiments included module ablation, multitask ablation, multi-frequency band comparison, and muscle fatigue experiments, which confirmed the superior performance of the model.

The remainder of this paper is structured as follows: The relevant work, including VAE, CAM, and MTL is introduced in Section 2. Data acquisition methods, data preprocessing, self-made dataset MI-EEMG, and the content of public datasets WAY-EEG-GAL and ESEMIT are described in Section 3. The 2M-hBCINet model proposed based on EEG and EMG signals is elucidated in Section 4 along with the extraction methods of EEG and EMG features, and the MTL strategy. The performance of the 2M-hBCINet model is validated and analyzed through extensive experiments in Section 5. Finally, the conclusions of this study are summarized and potential future directions are discussed in Section 6.

## 2 Related work

### 2.1 VAE

The VAE is a type of deep generative model introduced by Kingma and Welling in 2014, based on variational Bayes inference (Kingma and Welling, 2022). VAE uses two neural networks to establish two probabilistic density models (Rezende and Mohamed, 2016): one for the variational inference of the original input data, generating a variational probabilistic distribution of the latent variables, known as the inference network, and the other generates an approximate probabilistic distribution of the original data based on the generated variational distribution of the latent variables, known as the generative network. VAE is an unsupervised learning method (Pu et al., 2016), and through its powerful probabilistic generation capabilities and variational inference mechanisms, it can effectively capture the variability of bioelectrical signals (EEG, EMG) and extract key information features related to MI tasks. In recent years, VAE has been widely applied in the field of bioelectrical signal processing. For instance, Xia et al. used VAE to map signals to a latent variable distribution and regularized it in tasks involving EEG signal processing, capturing the uncertainty and variability within the signals. This representation in the latent space allows the model to effectively separate noise from useful features and preserve the key information of the signal during denoising and feature extraction (Xia et al., 2023). Chen et al. improved the feature extraction capability and reconstruction effect of EEG signals through VAE and applied a Gaussian prior distribution on the latent features, enabling VAE to capture the main characteristics of the input EEG signals (Chen et al., 2020). In this study, we extracted deep features from EEG and EMG signals using VAE to enhance the representational capacity of features for MI tasks.

### 2.2 CAM

The CAM is an attention mechanism designed to enhance the performance of convolutional neural networks (CNNs). Initially introduced by Jie et al., in 2017 within Squeeze-and-Excitation Networks (SENet), the core concept of the mechanism involves capturing the global features of channels through global pooling operations, followed by a lightweight self-gating mechanism to learn and recalibrate the importance weights of each channel (Jie et al., 2019). In practical applications, the CAM has been widely used across various research domains. For instance, Tang et al. incorporated a multiscale channel attention mechanism into their proposed architecture of a multi-scale channel attention CNN (MCA-CNN), effectively capturing complex features in the spectral domain (Tang et al., 2024). Wang et al. utilized the CAM to enable CNNs to thoroughly exploit the characteristic information of photoelectric peaks and Compton edges while suppressing background noise and interference, further enhancing the model performance (Wang et al., 2022). These studies indicate that the CAM significantly improves the sensitivity and robustness of CNNs to features. The CAM can achieve the goal of deep feature selection by increasing the weights of advantageous features and decreasing the weights of disadvantageous features (Srivastava et al., 2022), thereby optimizing the model’s training effects. In this study, we employed CAM to select and integrate the deep features of EEG and EMG, effectively enhancing the expressiveness and discriminability of the features.

### 2.3 MTL

MTL was initially proposed by Caruana (Caruana, 1997) as a method to improve data efficiency and reduce overfitting by sharing models to learn multiple tasks in parallel. MTL refers to the modeling of at least two tasks in a single deep-learning model (Zhou et al., 2018). By sharing some parameters, the MTL can effectively enhance the performance of each task and has been widely applied in the processing of bioelectrical signals. For instance, He et al. proposed a novel end-to-end multimodal multitask neural network model, and showed that the classification accuracy of the MTL approach improved by 4.8%, 4.4%, and 8.6% over single-task methods, respectively (He et al., 2022). In addition, Medhi et al. proposed a deep MTL approach to enhance the detection and classification of arrhythmias in electrocardiogram (ECG) signals. By jointly training multiple related tasks, MTL can capture the commonalities between tasks and improve the performance of each task through shared knowledge, thereby significantly improving the detection and classification of ECG arrhythmias (Medhi et al., 2023). In this study, we adopted the MTL approach to train our model, selecting tasks that included the primary task that is MI classification, and auxiliary tasks such as EEG reconstruction, EMG reconstruction, and feature metric learning tasks, thereby enhancing the model’s generalizability and accuracy.

## 3 Datasets and preprocessing

### 3.1 Datasets

In this study, we utilized a self-made dataset, MI-EEMG, and publicly available datasets from Mendeley Data, specifically the WAY-EEG-GAL and ESEMIT datasets. The following sections provide a detailed introduction regarding data collection and content information of the in-house dataset, and the content information of the public datasets.

#### 3.1.1 MI-EEMG

For this study, we collected EEG and EMG data from 14 participants performing MI and created an MI-EEMG dataset. The group consisted of six males (average age, 21.5 ± 3.2 years) and eight females (average age, 22.4 ± 4.6 years). All participants were right-handed, had normal vision, no motor impairments, and signed consent forms before the experiment. Participants were informed regarding the experimental procedures and precautions.

**FIGURE 1:**
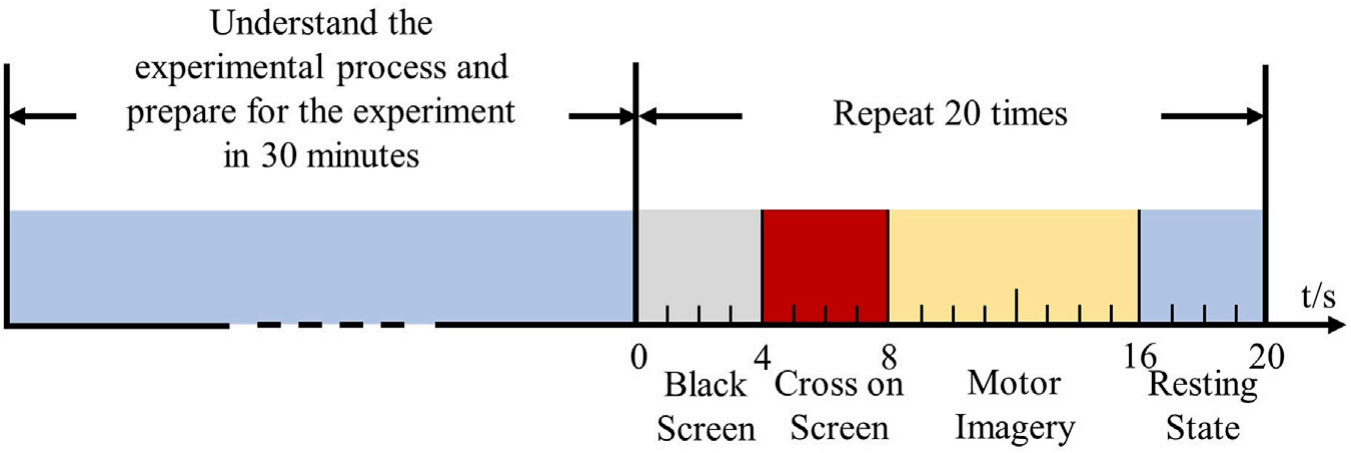
Experimental process of myoelectric electromyography acquisition. Fourteen participants were informed regarding the experimental procedure and prepared for the experiment within 30 min. They then performed MI tasks (left hand, right hand, and resting state) guided by cross signals on a screen, to complete the collection of electromyography (EMG) signals.

The MI-EEMG dataset included three types of MI actions: left hand, right hand, and resting state. Each subject underwent 30 trials of 10-s MI experiments, as shown in Figure 1. EEG electrodes were placed according to the international 10–20 system (Klem et al., 1999), with 32 channels and a sampling frequency of 500 Hz; four EMG electrodes were located on the left biceps (LB), left triceps (LT), right biceps (RB), and right triceps (RT), all with a sampling frequency of 500 Hz.

#### 3.1.2 WAY-EEG-GAL

The WAY-EEG-GAL dataset included data from 12 participants who were right-handed, had normal vision, and no motor impairments. During the EEG and EMG data collection process, the participants were instructed to reach out and grasp an object when prompted, lift it using their thumb and index finger, hold it for a few seconds, place it back on the support surface, release it, and finally return their hand to a specified resting position. EEG signals were captured using 32 channels from the international 10–20 system, and the EMG electrodes were positioned on the hands and arms. The experiment involved a total of 3,936 EEG and EMG data collections.

#### 3.1.3 ESEMIT

The ESEMIT dataset included data from 26 participants, including 10 males (average age, 25.4 ± 7.4 years) and 16 females (average age, 23 ± 3.2 years). All the participants were right-handed, had normal vision, and no motor impairments. The ESEMIT dataset includes three types of MI actions: left hand, right hand, and resting state. Each participant underwent a 4-min resting state and 15 min of MI induced by VR for collecting EEG and EMG data. EEG electrodes were placed according to the international 10–20 system, with 32 channels and a sampling frequency of 500 Hz; two EMG electrodes were located on the LB and RB, with a sampling frequency of 500 Hz.

### 3.2 Preprocessing

#### 3.2.1 EEG preprocessing

The original EEG signals were recorded in this study as shown in Equation 1:

$$X=\left(x_{1},x_{2},,x_{C_{1}}\right)\in R^{C_{1}\times L_{1}}$$ (1)

Where $C_{1}$ represents the number of channels for EEG acquisition ( $C_{1}=32$ in this study), $L_{1}$ denotes the number of samples, with $L_{1}=T\times f_{EEG}$ , T is the sampling time, and $f_{EEG}$ is the EEG sampling frequency.

EEG data are very weak bioelectrical signals and factors such as ECG signals, electromagnetic waves generated by power components, and inherent noise from the acquisition equipment can cause interference during data collection (Ferracuti et al., 2021). Therefore, it is necessary to preprocess EEG signals to eliminate interference and improve the signal-to-noise ratio. In this study, EEG preprocessing (Sun and Mou, 2023) included baseline correction, 50 Hz notch filtering, 4–50 Hz bandpass filtering, independent component analysis (Zhukov et al., 2000), and removal of EOG artifacts (ElSayed et al., 2021).

#### 3.2.2 EMG preprocessing

The original EMG signals were recorded in this study as shown in Equation 2:

$$Y=\left(y_{1},y_{2},,y_{C_{2}}\right)\in R^{C_{2}\times L_{2}}$$ (2)

Where $C_{2}$ represents the number of channels for EMG acquisition ( $C_{2}=4$ in this study), $L_{2}$ denotes the number of samples ( $L_{2}=T\times f_{EMG}$ **)**, T denotes the sampling time, and $f_{EMG}$ is the EMG sampling frequency.

Similar to EEG signals, EMG signals also require preprocessing to remove noise. This study employed a bandpass filter from 30 to 150 Hz to eliminate the influence of ECG signals, along with a 50 Hz notch filter to remove specific frequency noise.

## 4 Methods

**FIGURE 2:**
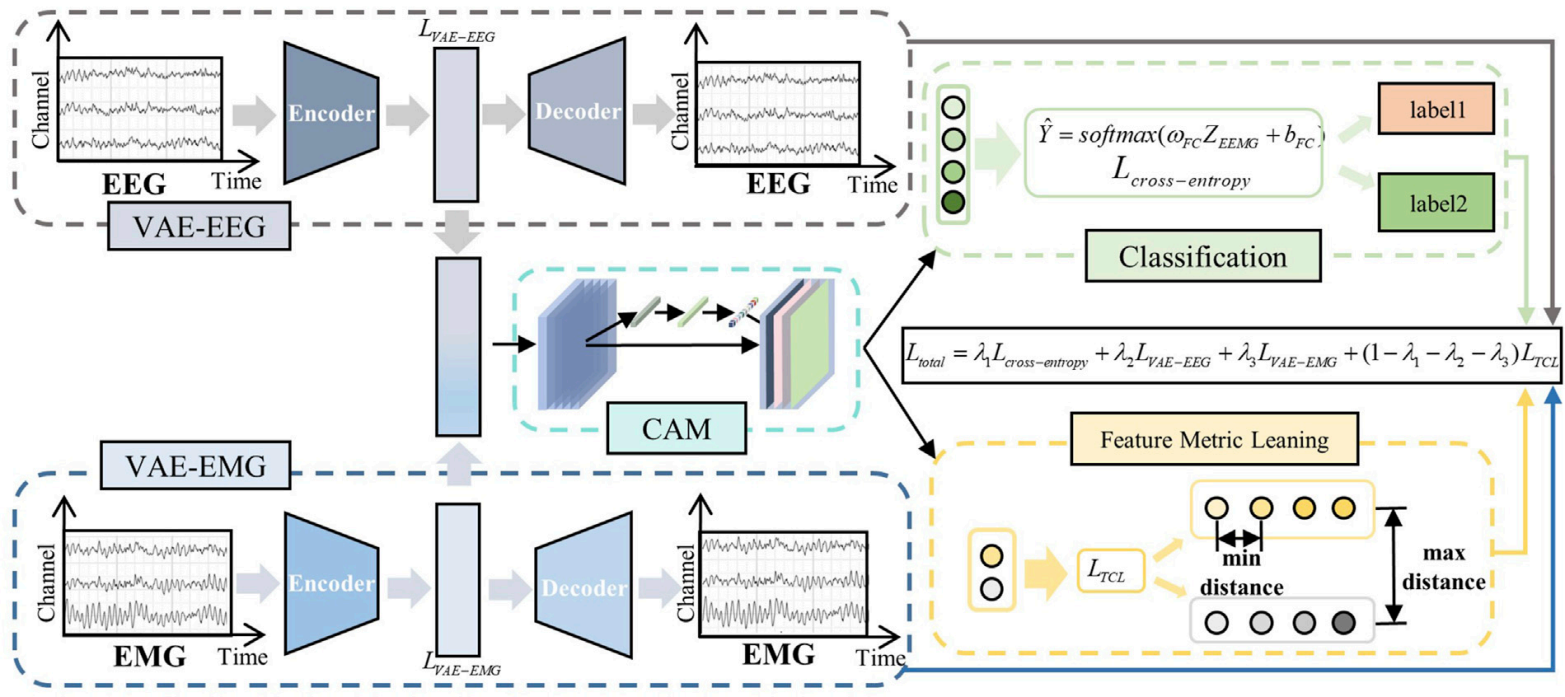
2M-hBCINet network structure. The system primarily includes three modules: the deep feature extraction module, the channel attention mechanism, and the multitask learning with loss functions. The VAE module efficiently integrates deep features from electroencephalogram (EEG) and electromyography (EMG) signals, while the CAM module intelligently allocates channel weights. Within the multitask framework, the primary task is motor imagery classification, and the auxiliary tasks include feature metric learning and EEG/EMG reconstruction. Different loss functions are applied to each task, including cross-entropy loss, triplet-center loss, and the VAE network’s loss function, and an overall loss function, Total Loss, is constructed.

Traditional MI classification task models tend to focus on the extraction of explicit features in the time and frequency domains; however, EEG and EMG signals possess a multitude of deep-level characteristics. Based on this, we designed a 2M-hBCINet model that can simultaneously extract deep features of EEG and EMG signals in an unsupervised manner and perform fusion. Additionally, an MTL approach was employed to enhance the performance and generalizability of the model, as shown in the network structure in Figure 2. The model consists of the following three parts: a deep feature extraction module, CAM, and MTL with loss functions.

### 4.1 Deep feature extraction module

#### 4.1.1 Encoding

##### 4.1.1.1 EEG electrode encoding

**FIGURE 3:**
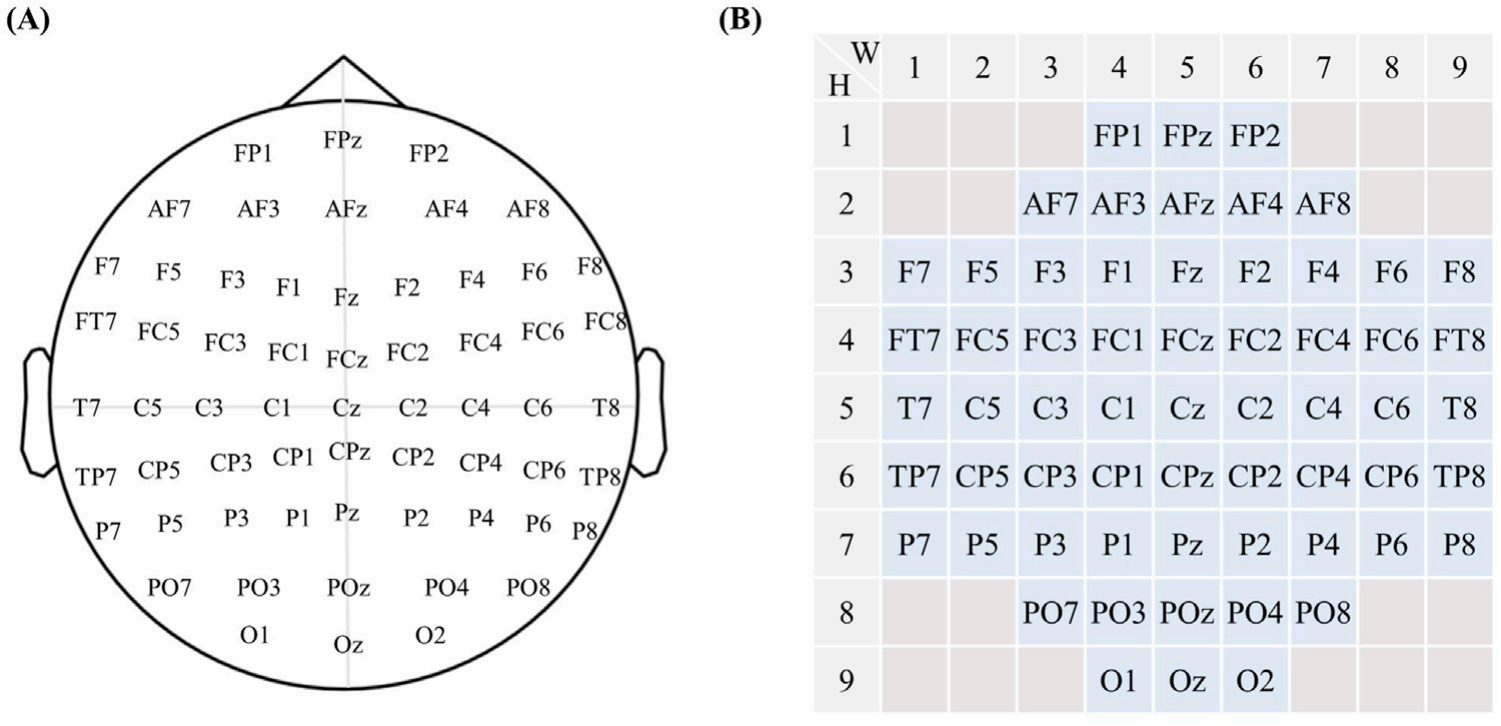
Electromyography electrode coding. **(A)** The current internationally accepted EEG electrode placement diagram. **(B)** Based on the internationally accepted EEG electrode placement system, the 10–20 system, electrodes have been numbered, with their positional coordinates denoted as $\left(W,H\right)$ , where W=9 and H=9 .

To better describe the EEG processing procedure, this study was based on the current internationally accepted EEG electrode placement rules 10–20 system. The brain electrodes were numbered, and the position coordinates of the electrodes were $\left(W,H\right)$ , ( W=9 , H=9 as shown in Figure 3 **)**. The spatial coordinates of the electrodes were $\left(W,H\right)$ , and the original spatiotemporal coordinates of the EEG were defined as Equation 3:

$$X_{EEG}\in R^{L_{EEG}\times H\times W}$$ (3)

Where $L=T\times f_{EEG}$ represents the number of sampling points, T is the sampling duration, and $f_{EEG}$ is the EEG sampling frequency.

##### 4.1.1.2 EMG electrode encoding

**FIGURE 4:**
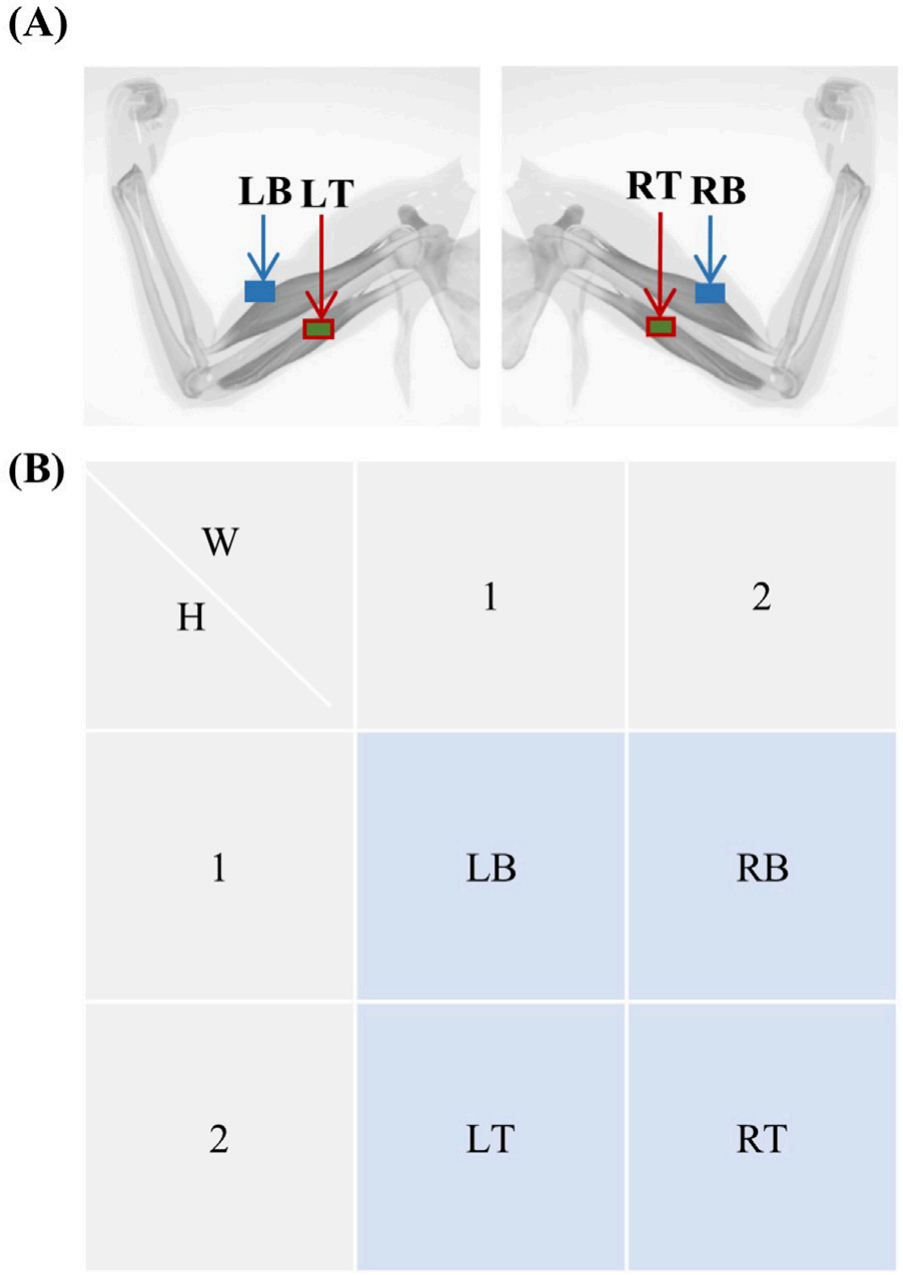
Electromyogram electrode coding. **(A)** Schematic diagram showing the locations of the left biceps (LB), left triceps (LT), right biceps (RB), and right triceps (RT). **(B)** Electrode numbering for electromyography, with electrode positional coordinates denoted as $\left(W,H\right)$ , where W=2 and H=2 .

As with the EEG electrodes, the EMG electrodes were assigned codes. Because of the small number of EMG electrodes, they were coded as follows: LB, LT, RB, and RT, as shown in Figure 4. The original spatiotemporal coordinates of the EMG are defined as Equation 4:

$$X_{EMG}\in R^{L_{EMG}\times 2\times 2}$$ (4)

where $L=T\times f_{EMG}$ represents the number of sampling points, T is the sampling duration, and $f_{EMG}$ denotes the EMG sampling frequency.

#### 4.1.2 Feature extraction

**FIGURE 5:**
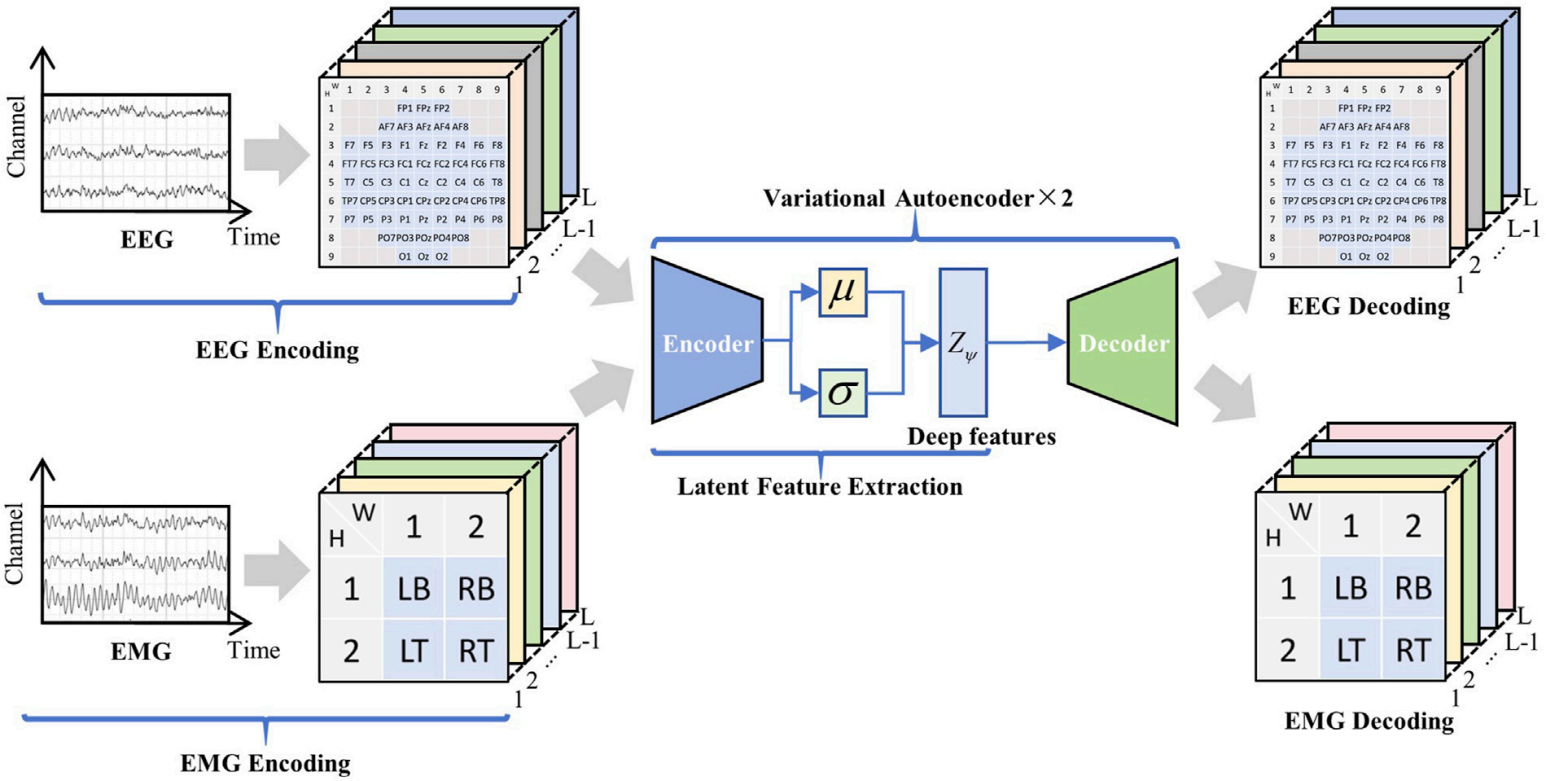
Deep feature extraction. Encoded EEG and EMG signals are fused through the VAE network to extract deep features $Z_{\psi }$ , where $\psi \in \left\{EEG,EMG\right\}$ .

As shown in Figure 5, this process consists of two steps: encoding and feature extraction of the bioelectrical signals. First, the collected bioelectric signals were encoded according to Equation 3 and Equation 4, to obtain $X_{\psi },\psi \in \left\{EEG,EMG\right\}$ . Then, $X_{\psi }$ was input into the VAE network for feature extraction, resulting in relevant deep features $Z_{\psi },\psi \in \left\{EEG,EMG\right\}$ .

Below is a detailed description of the process for extracting deep features from EEG and EMG signals using VAE. Let the input EEG and EMG signals be denoted as X , with its true distribution probability represented as $P_{\theta }\left(X\right)$ . The relevant latent features Z are obtained from X using a VAE, and the reconstructed data are denoted as $X^{\prime }$ . Then the inferential network can be expressed as $Q_{\phi }\left(Z|X\right)$ , and the generative network can be expressed as $P_{\theta }\left(Z\right)P_{\theta }\left(X^{\prime }\left|Z\right.\right)$ .

Because the potential feature Z is unobservable and cannot be solved directly, Z is estimated by calculating the lower bound. The true posterior distribution that the inference network could not determine is denoted as $P_{\theta }\left(Z|X\right)$ , and it is assumed that the inferred network $Q_{\phi }\left(Z|X\right)$ is a known distribution, and $Q_{\phi }\left(Z|X\right)$ is used to approximate the replacement of $P_{\theta }\left(Z|X\right)$ , such that both the VAE inferred and generated networks are known distributions. To make $Q_{\phi }\left(Z|X\right)$ as close to $P_{\theta }\left(Z|X\right)$ as possible, KL divergence is used to measure the degree of similarity between the two, and is minimized by optimizing the constraint parameters θ and ϕ . It can be expressed as shown in Equation 5:

$$\phi ,\theta =\arg\;\min\;D_{KL}\left(Q_{\phi }\left.\left(Z|X\right)\right\|P_{\theta }\left(Z|X\right)\right)=E_{Q_{\phi }\left(Z|X\right)}\left[1bQ_{\phi }\left(Z|X\right)-1bP_{\theta }\left(Z|X\right)\right]+1bP_{\theta }\left(X\right)$$ (5)

Noted as Equation 6:

$$L\left(\theta ,\phi ;X\right)=E_{Q_{\phi }\left(Z|X\right)}\left[1bQ_{\phi }\left(Z|X\right)-1bP_{\theta }\left(Z|X\right)\right]$$ (6)

It can be obtained as Equation 7:

$$1bP_{\theta }\left(X\right)=D_{KL}/(\left.Q_{\phi }\left(Z|X\right)\right\|P_{\theta }\left(Z|X\right)+L\left(\theta ,\phi ;X\right)$$ (7)

Since the KL divergence $D_{KL}\left(*\right)\geq 0$ is always true, therefore $1bP_{\theta }\left(X\right)\geq L\left(\theta ,\phi ;X\right)$ is always true; thus, the variational lower bound function of the marginal likelihood $1bP_{\theta }\left(X\right)$ of the input electrical signal $X_{\psi }$ can be obtained as $L\left(\theta ,\phi ;X\right)$ . Assuming $Q_{\phi }\left(Z|X\right)$ follows a normal distribution, the optimization objective for the inferred network is Equation 8:

$$\phi ,\theta =\underset{\phi ,\theta }{\arg\;\max}L\left(\theta ,\phi ;X\right)$$ (8)

To generate samples, the conditional distribution is generally a Bernoulli or Gaussian distribution whose probability density function is obtained using neural network computation. The optimization objective for the generative network is given in Equation 8.

The optimization objective of both the inferential and generative networks is to maximize the variational lower-bound function; therefore, the optimization objective of the VAE is to maximize the variational lower-bound function. Subsequently, the auxiliary parameter ε is introduced to transform the distribution of $Q_{\phi }\left(Z|X\right)$ to obtain $G_{\phi }\left(\varepsilon ,X\right)$ with $Z_{\psi }=G_{\phi }\left(\varepsilon ,X\right)$ , where $\varepsilon ∼p\left(\varepsilon \right)$ , and $p\left(\varepsilon \right)$ have a known marginal likelihood distribution. Assuming that $Q_{\phi }\left(Z_{\psi }|X_{\psi }\right)$ follows a normal distribution, the sampling of $Z_{\psi }$ can be described as Equation 9:

$$Z^{i}=\mu^{i}+\sigma^{i}\varepsilon^{i}$$ (9)

where μ represents the mean of the posterior probability and $\sigma^{2}$ represents the variance of the posterior probability. Subsequently, the variational lower bound can be simplified as Equation 10:

$$L\left(\theta ,\phi ;X\right)=-D_{KL}\left(Q_{\phi }\left.\left(Z|X\right)\right\|P_{\theta }\left(Z|X\right)\right)+\frac{1}{L}\sum_{l=1}^{L}1bP_{\theta }\left(X_{i}^{\prime }|Z^{i,j}\right)=\frac{1}{2}\sum \left[1b{\left(\sigma^{i}\right)}^{2}-{\left(\mu^{i}\right)}^{2}-{\left(\sigma^{i}\right)}^{2}+1\right]+1bP_{\theta }\left(X_{i}^{\prime }|Z^{i}\right)$$ (10)

The introduction of auxiliary parameters changes the relationship between Z and σ and μ from sampling calculation to numerical calculation, which can be optimized using the stochastic gradient descent method (Dai and Wipf, 2019). The mean and standard deviation of $P_{\theta }\left(X_{i}^{\prime }|Z^{i}\right)$ can be calculated using neural network, enabling the calculation of each term in the lower bound of the variational inference. Consequently, the structure of the VAE model can be established.

### 4.2 CAM

**FIGURE 6:**
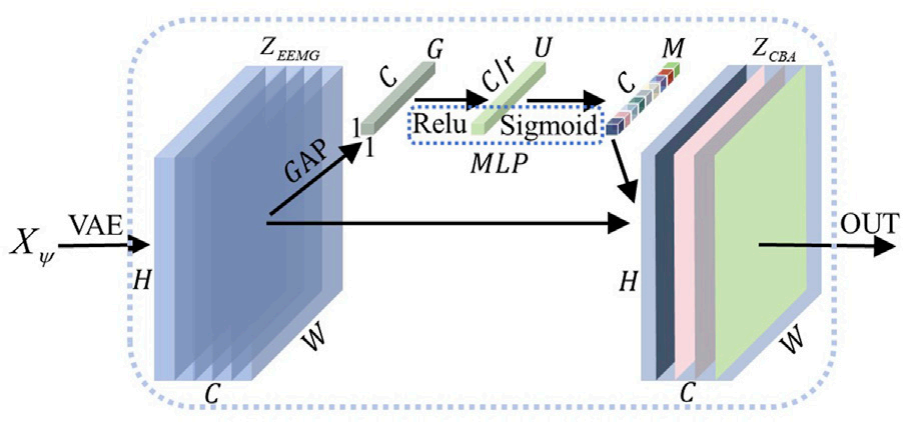
Channel attention mechanism. After feature extraction by the VAE module, the electrical signal X produces a feature map Z . Global average pooling compresses the spatial information of Z into a global feature value G . This global feature value G is then fed into a multi-layer perceptron (MLP) with ReLU and Sigmoid activation functions to compute attention weights $M_{c}$ for each channel. These weights are then applied to the original feature map to obtain the final feature map.

In this study, the CAM was used to select and fuse the extracted EEG and EMG deep features. This end-to-end trainable module enhances the network’s ability to focus on key information while suppressing extraneous information, thus improving the accuracy and generalization of the model. The detailed process for processing deep features using CAM is shown in Figure 6.

This module highlights useful EEG and EMG signal features. After the EEG and EMG signals pass through the VAE module, they obtain features denoted as $Z\in R^{H\times W\times C}$ . To capture the spatial information of each channel, a global average pooling (GAP) over the spatial dimensions of Z is required, which compresses the spatial information of each channel into a single global feature value, denoted as G . The dimension then becomes 1×1×C . The computation process for the c **-th** channel can be described as Equation 11:

$$G_{c}=\frac{1}{H\times W}\sum_{i=1}^{H}\sum_{j=1}^{W}z_{c}\left(i,j\right)$$ (11)

where $z_{c}\left(i,j\right)$ represents the feature of the channel c at position $\left(i,j\right)$ ;

The computational results are sent to a multilayer perceptron (MLP) for processing to capture the dependencies between channels. When the MLP contains two fully connected layers, the first layer maps the features to a lower dimension C/r and applies the ReLU activation function to introduce nonlinearity to obtain the output U . Assuming the weight matrix $W_{1}$ and the bias vector $b_{1}$ , the output U can be expressed as Equation 12:

$$U=ReLU\left(W_{1}\cdot G_{c}+b_{1}\right)$$ (12)

where the dimension of $W_{1}$ is C×C/r , and r is the dimensionality reduction coefficient used by MLP to decrease the computational effort by reducing the number of channels.

The second fully connected layer maps the vectors of the intermediate dimensions back to the original channel dimensions and applies a sigmoid activation function to restrict the weights to between 0 and 1 to obtain the channel attention weight matrix $M_{c}$ . Similar to the first layer, $M_{c}$ can be expressed as Equation 13:

$$M_{c}=Sigmoid\left(W_{2}\cdot U+b_{2}\right)$$ (13)

where the $W_{2}$ dimension is C/r×C and the output dimension of $M_{c}$ is guaranteed to be 1×1×C ;

At this time, the attention weight $M_{c}$ of each channel is obtained, which indicates the degree of “attention” of the model to each channel, and the weight is applied to the original feature map $Z_{c}$ , and the final feature map $\tilde{Z}$ is weighted by element-by-element multiplication of the feature map, as shown in Equation 14:

$$\tilde{Z}=M_{c}⊙Z$$ (14)

CAM realizes the effective utilization and enhancement of different channel information in the feature map through three steps: global information aggregation, interchannel dependency modeling, and attention weighting.

### 4.3 MTL

To enhance the classification accuracy of MI tasks and improve the model’s generalization capabilities (Ruder, 2017), this study incorporated a MTL approach to train the 2M-hBCINet. In this study, three learning objectives were set. The primary task was the MI classification task, and the auxiliary tasks included feature metric learning and EEG and EMG reconstruction. Feature metric learning aimed to minimize the distance between similar samples and maximize the distance between dissimilar samples by optimizing the metric criterion to learn more discriminative and expressive features, which indirectly improved the performance of the primary task.

Different loss functions were used for the different tasks in this study. For the primary task of MI classification and the auxiliary tasks of feature metric learning, EEG, and EMG reconstruction, the loss functions of cross-entropy loss, triplet-center loss, and VAE network were used, respectively, and the overall loss function was constructed as the total loss.

#### 4.3.1 Cross-entropy loss

To ensure that the classification results were close to the actual situation in the MI classification task, cross-entropy was adopted as the loss function. Cross-entropy loss was first introduced by Rubinstein in 1999 (Rubinstein, 1999), and it measures the discrepancy between the predicted and true distributions through simple calculations. The calculation is shown in Equation 15:

$$L_{cross-entropy}\left(y,\hat{y}\right)=-\sum_{k=1}^{k}y_{k}\log{\hat{y}}_{k},\left(k=0,1\right)$$ (15)

where y and $\hat{y}$ represent the true label and classification model prediction probabilities, respectively. The result of the MI classification task is the type with the highest probability.

#### 4.3.2 Triplet-center loss

In feature metric learning as the auxiliary task, the triplet-center loss (TCL) (He et al., 2018) was used as the loss function. TCL combines the advantages of triplet loss for interclass relationship processing with those of center loss for intraclass relationship processing and reduces certain computational complexities to effectively improve the accuracy of feature metric learning, which is calculated as in Equation 16

$$L_{TCL}=\sum_{i=1}^{M}\max\;\left(D\left(f_{i},c_{y^{i}}\right)+m-\min_{j\neq y^{i}}D\left(f\left(x^{i}\right),c_{j}\right),0\right)$$ (16)

Where, $y^{i}$ represents the label of the i **-th** type, $f\left(\cdot \right)$ represents the feature vector extracted in the neural network, $f_{i}$ represents the feature vector extracted by f from the i **-th** class, $c_{j}$ represents the center of the j **-th** class, and $D\left(\cdot \right)$ is the euclidean distance function is expressed as Equation 17:

$$D\left(f\left(x_{i}\right),c_{y^{i}}\right)=\frac{1}{2}{\left\|f\left(x_{i}\right)-c_{y^{i}}\right\|}_{2}^{2}$$ (17)

#### 4.3.3 TotaI loss

In this study, the abovementioned loss functions $L_{cross-entropy}$ and $L_{TCL}$ and the loss functions $L_{VAE-EEG}$ and $L_{VAE-EMG}$ of the VAE network were combined to obtain the final loss function:

$$L_{total}=\lambda_{1}L_{cross-entropy}+\lambda_{2}L_{VAE-EEG}+\lambda_{3}L_{VAE-EMG}+\left(1-\lambda_{1}-\lambda_{2}-\lambda_{3}\right)L_{TCL}$$ (18)

where, $\lambda_{1}$ , $\lambda_{2}$ , and $\lambda_{3}$ represent the hyperparameters for the contributions of models $L_{cross-entropy}$ , $L_{VAE-EEG}$ , and $L_{VAE-EMG}$ , respectively, satisfying the condition $\lambda_{1}+\lambda_{2}+\lambda_{3}\leq 1$ . To determine the hyperparameters in Equation 18, a preliminary study was conducted using a grid-search algorithm. The sets of parameters $\lambda_{1}$ , $\lambda_{2}$ , and $\lambda_{3}$ in the grid search algorithm were defined as {0.1, 0.2, 0.3, 0.4, 0.5, 0.6, 0.7}. The experimental results indicate that $\lambda_{1}$ , $\lambda_{2}$ , and $\lambda_{3}$ should be set to 0.7, 0.1, and 0.1, respectively. In addition, to complete the multitask ablation experiments, the parameters for two-task and three-task collaborative training were investigated. The parameters for the two-task model 2M-hBCINet-CEEGR were $\lambda_{1}=0.7$ and $\lambda_{2}=0.3$ , while the parameters for the model 2M-hBCINet-CEMGR were $\lambda_{1}=0.7$ and $\lambda_{3}=0.3$ . The parameter for the model 2M-hBCINet-CF was $\lambda_{1}=0.7$ . For the three-task model 2M-hBCINet-CEEMGR, the parameters were $\lambda_{1}=0.6$ , $\lambda_{2}=0.2$ , and $\lambda_{3}=0.2$ . The parameters for the model 2M-hBCINet-CEEGRF were $\lambda_{1}=0.6$ and $\lambda_{2}=0.2$ , and for the model 2M-hBCINet-CEMGRF, the parameters were $\lambda_{1}=0.6$ and $\lambda_{3}=0.2$ .

### 4.4 2M-hBCINet

To better accomplish MI tasks, we explored, selected, and integrated the deep features of EEG and EMG signals, and designed a 2M-hBCINet model.

First, the raw EEG and EMG signals were encoded, and deep features were extracted using the VAE network. The structure of the VAE network is shown in Figure 2, which consists of an encoder and a decoder. The extraction process of deep spatio-temporal feature $X_{\psi },\psi \in \left\{EEG,EMG\right\}$ is shown in Figure 5. After completing $X_{\psi }$ extraction, CAM was used to combine, select, and assign relevant weights to the features, as shown in Figure 6, which effectively improved the utilization of potential features. In addition, the 2M-hBCINet model adopts a MTL approach for training. The primary task designed for this study was the MI classification task, whereas the auxiliary tasks included a feature metric learning task and reconstruction tasks for both EEG and EMG, along with the relevant loss functions, $L_{cross-entropy}$ and $L_{TCL}$ . These were combined with the VAE network loss functions, $L_{VAE-EEG}$ and $L_{VAE-EMG}$ to obtain the overall loss function $L_{total}$ .

Subsequently, joint training of the 2M-hBCINet model was performed by updating the parameters in the VAE network, CAM, weight parameters, and bias values in the fully connected (FC) layer in each training session, as shown in Algorithm 1. After the fully connected layer, the predicted values for MI classification were calculated using Equation 19. $Z_{EEG}$ represents the EEG potential features extracted by the ${VAE}_{EEG}$ network, $Z_{EMG}$ represents the EMG potential features extracted by the ${VAE}_{EMG}$ network, $Z_{EEMG}$ represents the features after fusing EEG and EMG, and $Z_{CBA}$ denotes the features after selection, fusion, and weighting by the CAM. The 2M-hBCINet model used the joint loss function $L_{total}$ as the loss function.

$$\hat{Y}=softmax\;\left(\omega_{FC}Z_{EEMG}+b_{FC}\right)$$ (19)

During the backpropagation process of the 2M-hBCINet model, the CAM and deep feature extraction module were updated synchronously. The specific process is as follows:

In the CAM, the gradient $\frac{\partial total\left(Y,\hat{Y}\right)}{\partial \hat{Y}}$ of the parameters of the fully connected layer is solved first, the gradient $\frac{\partial \hat{Y}}{\partial {Z_{FC}}^{t+1}}$ of the fully connected layer acting on CAM is calculated next, and finally, the gradient $\frac{\partial {Z_{FC}}^{t+1}}{\partial {{\;\omega }_{CBA}}^{t}}$ of the parameters of the entire CAM is calculated. According to the chain rule, the gradient $\nabla {\omega_{CBA}}^{t+1}=\frac{\partial total\left(Y,\hat{Y}\right)}{\partial \hat{Y}}\cdot \frac{\partial \hat{Y}}{\partial {Z_{FC}}^{t+1}}\cdot \frac{\partial {Z_{FC}}^{t+1}}{\partial {\;\omega_{CBA}}^{t}}$ of the CAM and the corresponding fully connected layer parameters can be obtained. Subsequently, the CBA network parameters are updated according to ${{\tilde{\omega }}_{CBA}}^{t+1}={\omega_{CBA}}^{t}-\eta_{CBA}\nabla {\;\omega_{CBA}}^{t+1}$ to complete the CAM parameter update.

For gradient extraction of parameters of the VAE module, the gradient $\frac{\partial {Z_{FC}}^{t+1}}{\partial {\;Z_{CBA}}^{t+1}}$ of the CAM parameters is solved, following which the gradients $\frac{\partial {Z_{CBA}}^{t+1}}{\partial {\;\omega_{EEG}}^{t}}$ and $\frac{\partial {Z_{CBA}}^{t+1}}{\partial {\;\omega_{EMG}}^{t}}$ of the ${VAE}_{EEG}$ and ${VAE}_{EMG}$ networks are computed respectively. According to the chain rule, the gradients $\nabla {\omega_{EEG}}^{t+1}=\frac{\partial total\left(Y,\hat{Y}\right)}{\partial \hat{Y}}\cdot \frac{\partial \hat{Y}}{\partial {Z_{FC}}^{t+1}}\cdot \frac{\cdot {Z_{FC}}^{t+1}}{\partial {\;Z_{CBA}}^{t+1}}\cdot \frac{\partial {Z_{CBA}}^{t+1}}{\partial {\;Z_{EEMG}}^{t+1}}\cdot \frac{\partial {\;Z_{EEMG}}^{t+1}}{\partial {\;\omega_{EEG}}^{t}}$ and $\nabla {\omega_{EMG}}^{t+1}=\frac{\partial total\left(Y,\hat{Y}\right)}{\partial \hat{Y}}\cdot \frac{\partial \hat{Y}}{\partial {Z_{FC}}^{t+1}}\cdot \frac{\partial {Z_{FC}}^{t+1}}{\partial {\;Z_{CBA}}^{t+1}}\cdot \frac{\partial {Z_{CBA}}^{t+1}}{\partial {\;Z_{EEMG}}^{t+1}}\cdot \frac{\partial {\;Z_{EEMG}}^{t+1}}{\partial {\;\omega_{EMG}}^{t}}$ of the VAE network parameters and the full connection layer parameters can be obtained. After that, the ${VAE}_{EEG}$ , ${VAE}_{EMG}$ network parameters are updated according to ${{\tilde{\omega }}_{EEG}}^{t+1}={\omega_{EEG}}^{t}-\eta_{VAE-EEG}\nabla {\;\omega_{EEG}}^{t+1}$ , ${{\tilde{\omega }}_{EMG}}^{t+1}={\omega_{EMG}}^{t}-\eta_{VAE-EMG}\nabla {\;\omega_{EMG}}^{t+1}$ to complete a VAE module parameter update.

After updating the CAM and VAE module, the parameters of the fully connected layer are updated according to ${{\tilde{\omega }}_{FC}}^{t+1}={\omega_{FC}}^{t}-\eta_{FC}\nabla {\;\omega_{FC}}^{t+1}$ such that it can completely learn the selected deep features.

Algorithm 1 2M-hBCINet.

Require: Bioelectric signal $X_{\psi },\psi \in \left\{EEG,EMG\right\}$ ;
    The motor imagery task corresponds to the label Y;
Ensure: parameters of VAE: $\omega_{EEG},\omega_{EMG}$ ; parameters of CBA: $\omega_{CBA}$ ; parameters of FC: $\omega_{FC}$ ;
1. for t=1;t<⁡max⁡;t++

1. ${Z_{EEG}}^{t+1}={VAE}_{EEG}\left(X_{EEG},{\omega_{EEG}}^{t}\right)$

2. ${Z_{EMG}}^{t+1}={VAE}_{EMG}\left(X_{EMG},{\omega_{EMG}}^{t}\right)$

3. ${Z_{EEMG}}^{t+1}={Z_{EEG}}^{t+1},{Z_{EMG}}^{t+1}$

4. ${Z_{CBA}}^{t+1}=CBA\left({Z_{EEMG}}^{t+1},{\omega_{CBA}}^{t}\right)$

5. $\hat{Y}=FC\left({Z_{CBA}}^{t+1},{\omega_{FC}}^{t}\right)$

6. $\nabla {\omega_{EEG}}^{t+1}=\frac{\partial total\left(Y,\hat{Y}\right)}{\partial \hat{Y}}\cdot \frac{\partial \hat{Y}}{\partial {Z_{FC}}^{t+1}}\cdot \frac{\partial {Z_{FC}}^{t+1}}{\partial {\;Z_{CBA}}^{t+1}}\cdot \frac{\partial {Z_{CBA}}^{t+1}}{\partial {\;Z_{EEMG}}^{t+1}}\cdot \frac{\partial {\;Z_{EEMG}}^{t+1}}{\partial {\;\omega_{EEG}}^{t}}$

7. $\nabla {\omega_{EMG}}^{t+1}=\frac{\partial total\left(Y,\hat{Y}\right)}{\partial \hat{Y}}\cdot \frac{\partial \hat{Y}}{\partial {Z_{FC}}^{t+1}}\cdot \frac{\partial {Z_{FC}}^{t+1}}{\partial {\;Z_{CBA}}^{t+1}}\cdot \frac{\partial {Z_{CBA}}^{t+1}}{\partial {\;Z_{EEMG}}^{t+1}}\cdot \frac{\partial {\;Z_{EEMG}}^{t+1}}{\partial {\;\omega_{EMG}}^{t}}$

8. ${{\tilde{\omega }}_{EEG}}^{t+1}={\omega_{EEG}}^{t}-\eta_{VAE-EEG}\nabla {\;\omega_{EEG}}^{t+1}$

9. ${{\tilde{\omega }}_{EMG}}^{t+1}={\omega_{EMG}}^{t}-\eta_{VAE-EMG}\nabla {\;\omega_{EMG}}^{t+1}$

10. ${{\nabla \omega }_{CBA}}^{t+1}=\frac{\partial total\left(Y,\hat{Y}\right)}{\partial \hat{Y}}\cdot \frac{\partial \hat{Y}}{\partial {Z_{FC}}^{t+1}}\cdot \frac{\partial {Z_{FC}}^{t+1}}{\partial {\;\omega_{CBA}}^{t}}$

11. ${{\tilde{\omega }}_{CBA}}^{t+1}={\omega_{CBA}}^{t}-\eta_{CBA}\nabla {\;\omega_{CBA}}^{t+1}$

12. ${{\nabla \omega }_{FC}}^{t+1}=\frac{\partial total\left(Y,\hat{Y}\right)}{\partial \hat{Y}}\cdot \frac{\partial \hat{Y}}{\partial {Z_{FC}}^{t}}$

13. ${{\tilde{\omega }}_{FC}}^{t+1}={\omega_{FC}}^{t}-\eta_{FC}\nabla {\;\omega_{FC}}^{t+1}$

14. end for

## 5 Experiment and discussion

### 5.1 Experimental setting

#### 5.1.1 Evaluation methods

Common machine learning evaluation metrics were selected for this study to serve as evaluation indicators, with the performance of the 2M-hBCINet model described from various perspectives. The evaluation indicators included accuracy, precision, recall, and F1-score. The calculation methods are shown in Equations 20–23: Model performance was assessed by plotting the receiver operating characteristic (ROC) curve and calculating the area under the curve (AUC), known as the ROC-AUC.

$$Accuracy=\left(TP+TN\right)/\left(TP+TN+FP+FN\right)$$ (20)

$$Precision=TP/\left(TP+FP\right)$$ (21)

$$Recall=TP/\left(TP+FN\right)$$ (22)

$$F1-score=\left(2\times Precision\times Recall\right)/\left(Precision+Recall\right)$$ (23)

where TP , FP , TN , and FN represent true positive, false positive, true negative, and false negative, respectively.

#### 5.1.2 Experimental methodology (LOOCV)

The LOOCV method (Walter et al., 2013) was employed to validate the performance of the 2M-hBCINet model. In the LOOCV method, one sample is extracted from the dataset to serve as the validation set for testing at each iteration until all samples are used as the validation set. The number of validations correspond to the number of samples, and the validation results are expressed as the average of all experimental outcomes, as indicated by Equation 24. The advantages of the LOOCV method include its ability to fully utilize data in small sample sizes for MI and to effectively prevent overfitting, thereby enabling a comprehensive assessment of the model’s generalization ability.

$$e_{M}=\frac{1}{N}\sum_{n=1}^{N}{err}_{M}\left(2M-hBCINet\left(x_{n}\right),y_{n}\right)$$

$$M\in \left\{Accuracy,Precision,Recall.F1-score,ROC-AUC\right\}$$ (24)

Where N represents the number of samples, n represents the index of the selected sample, $x_{n}$ represents the samples, $y_{n}$ represents the corresponding labels of the samples, M represents the selected evaluation indices, ${err}_{M}\left(\cdot \right)$ denotes the performance assessment results of the 2M-hBCINet model using different evaluation indices, and $e_{M}$ represents the validation results obtained for the corresponding evaluation metrics using the LOOCV method.

#### 5.1.3 Parameter setting

This study used parameter settings for three models: ${VAE}_{EEG}$ , ${VAE}_{EMG}$ , and total loss function. A symmetrical structure consisting of four convolutional layers was utilized for encoding and decoding in both ${VAE}_{EEG}$ and ${VAE}_{EMG}$ . Each layer was composed of convolutional kernels of sizes 3×3×128 , 3×3×256 , 3×3×256, and 3×3×512 , with a stride of 1. Hyperparameters $\lambda_{1}$ , $\lambda_{2}$ , and $\lambda_{3}$ were set to 0.7, 0.1, and 0.1, respectively, for the total loss function.

### 5.2 Experiment and discussion

We performed several experiments involving the LOOCV method on the self-made MI-EEMG dataset and the public datasets WAY-EEG-GAL and ESEMIT. These experiments include module ablation studies, comparisons of MTL, multi-band comparison experiments, and muscle fatigue experiments.

#### 5.2.1 Module ablation experiment

To validate the effectiveness of each module of the proposed 2M-hBCINet model, an ablation study was conducted using the MI-EEMG, WAY-EEG-GAL, and ESEMIT datasets. LOOCV was used as the validation method. The validated models included model ${VAE}_{EEG}-CBA$ without the module ${VAE}_{EMG}$ , model ${VAE}_{EMG}-CBA$ without the module ${VAE}_{EEG}$ , model ${VAE}_{EEG}-{VAE}_{EMG}$ without the module CBA, and the complete 2M-hBCINet model.

**FIGURE 7:**
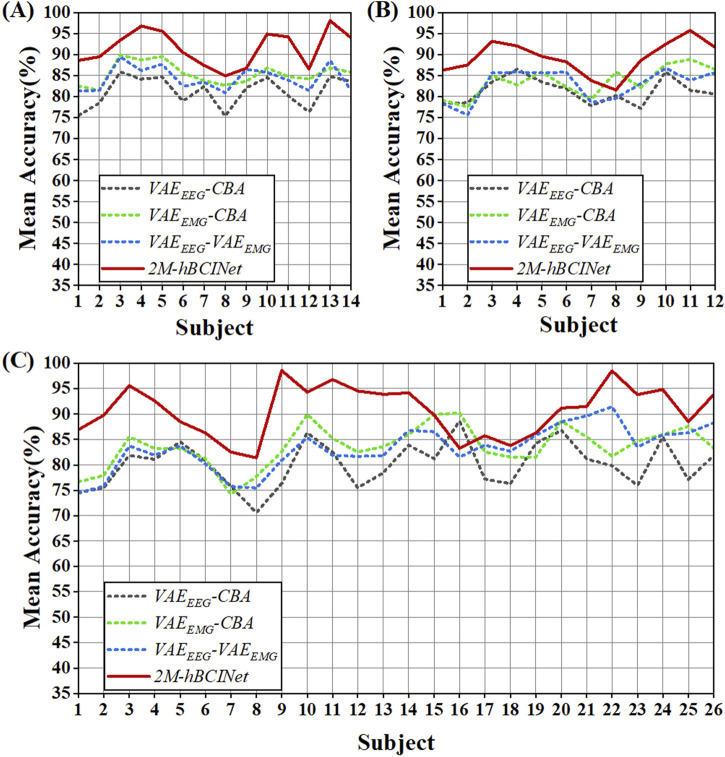
Statistical results of module ablation experiment. **(A)** In the MI-EEMG dataset, 70 motor imagery classification experiments were performed for each model (14 participants × 5 trials). Compared to models missing certain modules, the 2M-hBCINet model showed an accuracy improvement of 6.1%–10.3%. **(B)** In the WAY-EEG-GAL dataset, 60 motor imagery classification experiments were performed for each model (12 participants × 5 trials). The 2M-hBCINet model demonstrated an increase of 5.7%–8.0% in accuracy over models with missing modules. **(C)** In the ESEMIT dataset, 130 motor imagery classification experiments were performed for each model (26 participants × 5 trials). The 2M-hBCINet model achieved an accuracy enhancement of 7.1%–10.5% compared to models without the respective modules.

**FIGURE 8:**
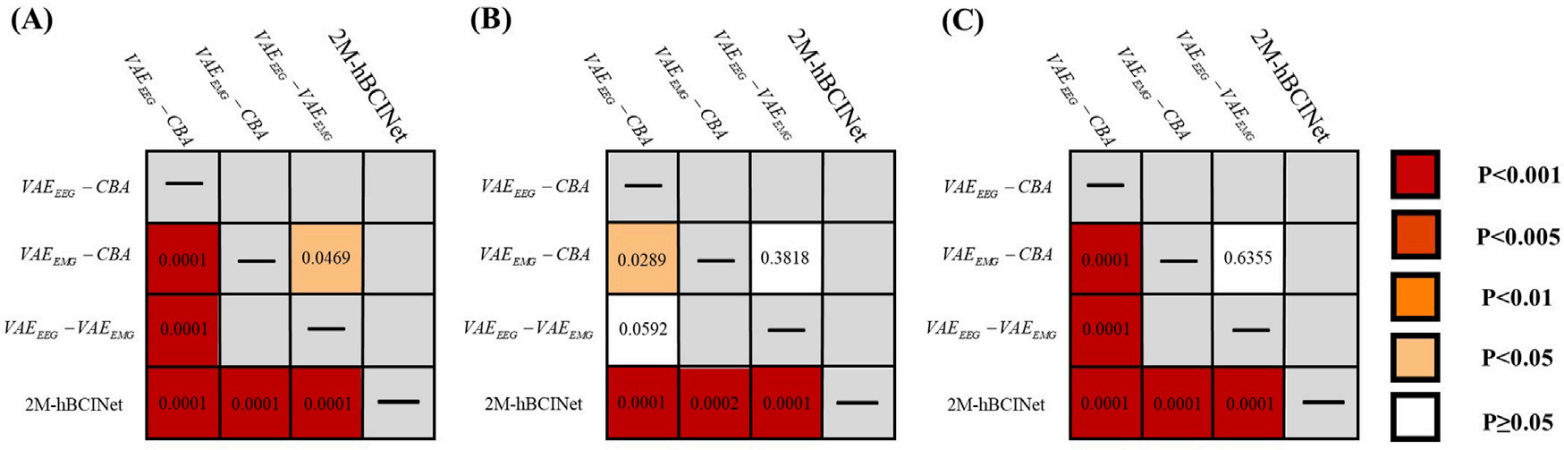
Significance analysis of each model in module ablation experiment. **(A)** The *t*-test results for each model in the MI-EEMG dataset are shown in figure. **(B)** The *t*-test results for each model in the WAY-EEG-GAL dataset are shown in figure. **(C)** The *t*-test results for each model in the ESEMIT dataset are shown in the figure.

In the MI-EEMG dataset, 70 MI classification experiments were conducted for each model (14 participants × 5 trials). Figure 7A illustrates the performance of each participant in this experiment, and Figure 8A presents the significance analysis results for each model. In the WAY-EEG-GAL dataset, 60 MI classification experiments were performed for each model (12 participants × 5 trials). Figure 7B shows the performance of each participant in the experiment, and Figure 8B shows the significance analysis results for each model. In the ESEMIT dataset, 130 MI classification experiments were performed for each model (26 participants × 5 trials). Figure 7C shows the performance of each participants in this experiment, and Figure 8C displays the significance analysis results for each model. The statistical results of the experiments for each model across all the datasets are presented in Table 1.

**TABLE 1:** Module ablation experiment.

Dataset	Model	Accuracy (%)	Precision	Recall	F1-score	ROC-AUC
MI-EEMG	${VAE}_{EEG}-CBA$	81.2	82.312	81.543	81.926	80.251
	${VAE}_{EMG}-CBA$	85.4	88.553	88.672	88.612	81.216
	${VAE}_{EEG}-{VAE}_{EMG}$	84.3	87.771	86.528	87.145	80.115
	2M-hBCINet	91.5	93.546	93.648	93.596	85.618
WAY-EEG-GAL	${VAE}_{EEG}-CBA$	81.3	83.562	81.796	82.700	78.562
	${VAE}_{EMG}-CBA$	83.6	84.523	84.196	84.359	79.653
	${VAE}_{EEG}-{VAE}_{EMG}$	82.9	84.128	85.963	85.036	78.956
	2M-hBCINet	89.3	91.256	90.568	90.911	87.635
ESEMIT	${VAE}_{EEG}-CBA$	80.1	82.617	81.152	81.878	78.156
	${VAE}_{EMG}-CBA$	83.5	85.618	84.612	85.112	81.624
	${VAE}_{EEG}-{VAE}_{EMG}$	83.2	85.946	85.946	85.946	82.653
	2M-hBCINet	90.6	93.612	92.584	93.095	96.325

Table 1 shows that the accuracy of the models missing various modules decreased by 6.1%–10.3%, 5.7%–8.0%, and 7.1%–10.5% compared to the 2M-hBCINet model. The precision, recall, and F1-score also decreased, demonstrating the necessity of each submodule. Furthermore, in both the MI-EEMG and ESEMIT datasets, the model ${VAE}_{EMG}-CBA$ achieved better performance than ${VAE}_{EEG}-{VAE}_{EMG}$ and ${VAE}_{EEG}-CBA$ . This indicates that the features obtained from EMG signals provide greater expressiveness for MI tasks and allow for better representation than features obtained from EEG signals.

In the significance analysis presented in Figure 8, the p-values are categorized into five levels: p≥0.05 , p<0.05 , p<0.01 , p<0.005 , and p<0.001; statistical significance was set at p<0.05 , with smaller p-values indicating greater significance and p≥0.05 indicating that the difference between the two models was not statistically significant. A statistically significant improvement in the accuracy of the 2M-hBCINet model compared to other models missing various modules indicated a notable difference, and demonstrated the significant superiority of the 2M-hBCINet model.

#### 5.2.2 MTL comparison experiments

**FIGURE 9:**
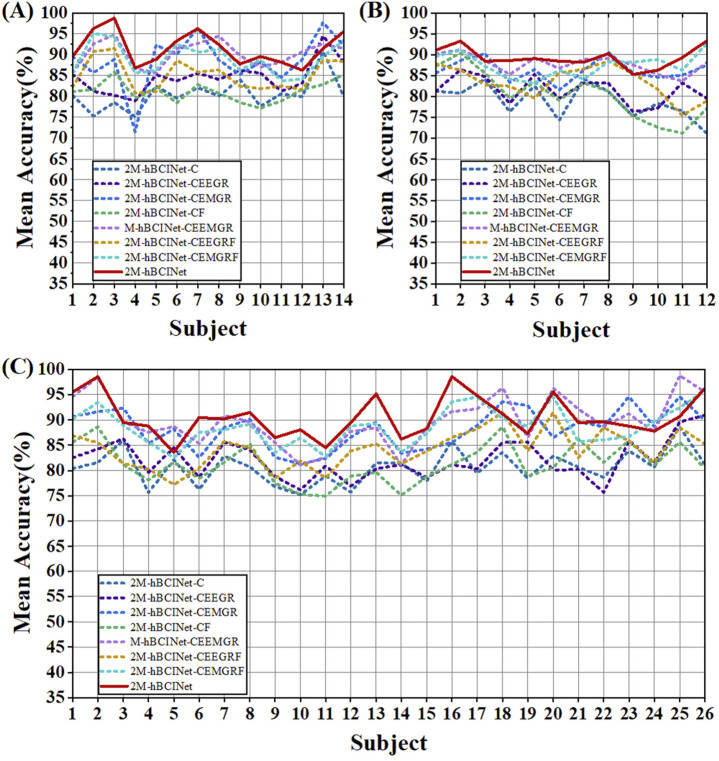
Statistical results of multitask learning comparison experiments. The models evaluated include single-task model 2M-hBCINet-C; two-task models 2M-hBCINet-CEEGR, 2M-hBCINet-CEMGR, and 2M-hBCINet-CF; three-task models 2M-hBCINet-CEEMGR, 2M-hBCINet-CEEGRF, and 2M-hBCINet-CEMGRF; and the four-task model 2M-hBCINet. **(A)** In the MI-EEMG dataset, 70 motor imagery classification experiments (14 participants × 5 trials) were performed to test each learning approach. The 2M-hBCINet model showed an average accuracy improvement of 1.2%–11.0% compared to other models. **(B)** In the WAY-EEG-GAL dataset, 60 motor imagery classification experiments were performed for each model (12 participants × 5 trials). The 2M-hBCINet model achieved an average accuracy increase of 1.5%–10.5% compared to other models. **(C)** In the ESEMIT dataset, 130 motor imagery classification experiments (26 participants × 5 trials) were conducted for each learning approach. The 2M-hBCINet model demonstrated an average accuracy enhancement of 1.7%–10.0% compared to other models.

**FIGURE 10:**
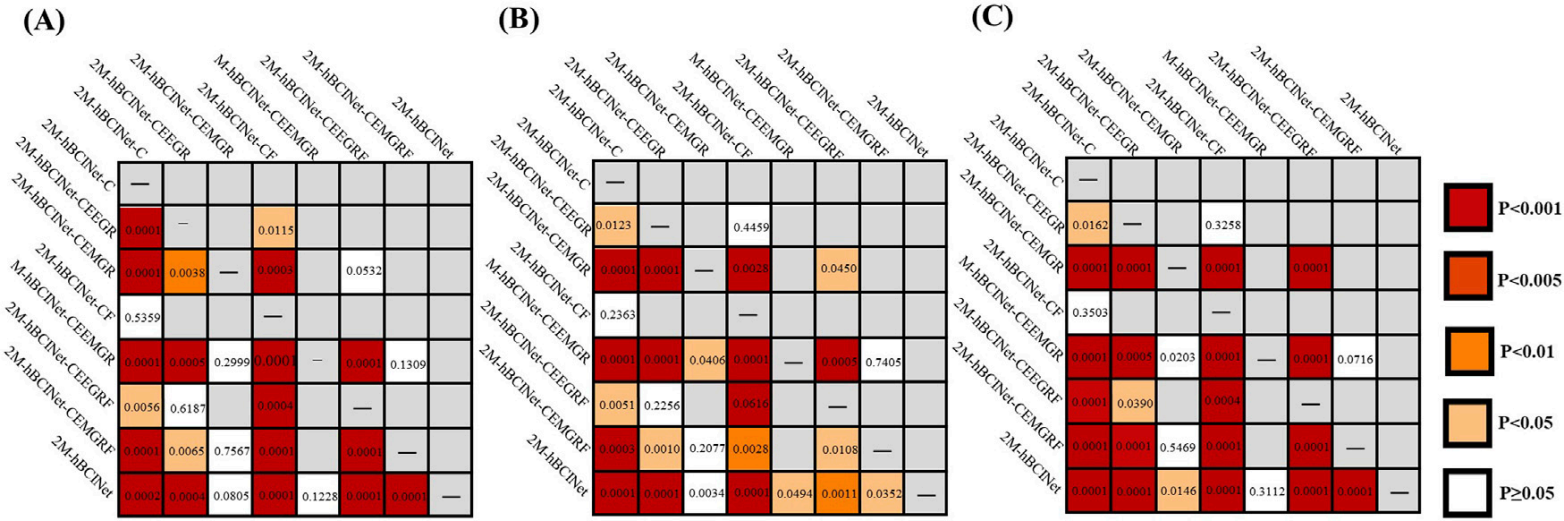
Significance analysis of each model in multitask learning comparison experiment. **(A)** In the MI-EEMG dataset, the *t*-test results for each model are depicted. **(B)** In the WAY-EEG-GAL dataset, the *t*-test results for each model are shown. **(C)** In the ESEMIT dataset, the *t*-test results for each model are shown.

To validate the effectiveness of the MTL approach in the proposed 2M-hBCINet model, comparative experiments were performed using the MI-EEMG, WAY-EEG-GAL, and ESEMIT datasets, with LOOCV as the validation method. The validated models included the single-task model 2M-hBCINet-C, the two-task models 2M-hBCINet-CEEGR, 2M-hBCINet-CEMGR, and 2M-hBCINet-CF, as well as the three-task models 2M-hBCINet-CEEMGR, 2M-hBCINet-CEEGRF, and 2M-hBCINet-CEMGRF, and the four-task model 2M-hBCINet. In this context, C, EEGR, EMGR, and F denote MI classification (primary task), EEG reconstruction, EMG reconstruction, and feature metric learning (auxiliary tasks), respectively. The dataset and number of experiments used in this study were consistent with those used in the ablation study. Figure 9 illustrates the experimental performance of the participants across different datasets, and Figure 10 presents the significance analysis results for each model, with A, B, and C corresponding to the three types of datasets. The experimental results are presented in Table 2.

**TABLE 2:** Multitask learning comparison experiment.

Dataset	Model	Accuracy (%)	Precision	Recall	F1-score	ROC-AUC
MI-EEMG	2M-hBCINet-C	80.5	82.617	82.157	82.386	80.654
	2M-hBCINet-CEEGR	84.5	85.617	86.023	85.820	83.512
	2M-hBCINet-CEMGR	88.6	90.235	90.845	90.539	87.684
	2M-hBCINet-CF	81.2	83.157	83.698	83.427	80.951
	M-hBCINet-CEEMGR	90.3	91.592	92.574	92.080	88.325
	2M-hBCINet-CEEGRF	85.2	88.561	89.624	89.089	84.158
	2M-hBCINet-CEMGRF	89.1	90.238	91.687	90.957	88.637
	2M-hBCINet	91.5	93.546	93.648	93.597	89.651
WAY-EEG-GAL	2M-hBCINet-C	78.8	79.562	80.752	80.153	76.532
	2M-hBCINet-CEEGR	81.5	81.968	82.345	82.156	79.635
	2M-hBCINet-CEMGR	86.3	87.628	86.355	86.987	84.523
	2M-hBCINet-CF	80.5	80.457	80.354	80.405	76.428
	M-hBCINet-CEEMGR	87.8	88.624	89.524	89.072	84.632
	2M-hBCINet-CEEGRF	83.4	84.529	83.524	84.023	80.528
	2M-hBCINet-CEMGRF	87.5	87.635	88.352	87.992	84.638
	2M-hBCINet	89.3	91.452	90.385	90.915	94.852
ESEMIT	2M-hBCINet-C	80.6	81.215	82.365	81.786	78.952
	2M-hBCINet-CEEGR	82.1	83.914	84.561	84.236	81.524
	2M-hBCINet-CEMGR	88.2	89.226	88.954	89.090	86.321
	2M-hBCINet-CF	81.3	82.645	82.648	82.646	78.514
	M-hBCINet-CEEMGR	89.9	90.621	91.584	91.100	86.952
	2M-hBCINet-CEEGRF	84.2	86.324	85.962	86.143	82.352
	2M-hBCINet-CEMGRF	88.7	89.452	90.236	89.842	86.325
	2M-hBCINet	90.6	93.612	92.584	93.095	96.325

Table 2 shows that the 2M-hBCINet model, which employs all tasks, achieved an average accuracy improvement of 1.2%–11.0%, 1.5%–10.5%, and 1.7%–10.0% with the MI-EEMG, WAY-EEG-GAL, and ESEMIT datasets, respectively, compared to other models. Additionally, the precision, recall, F1-score, and ROC-AUC evaluation metrics showed improvements, demonstrating the effectiveness of each task during the model training process. This enhancement is attributed to the strong correlation between the selected EEG reconstruction, EMG reconstruction, and feature metric learning tasks, and the primary task of MI classification. The collaborative training process allows for mutual reinforcement and complementary learning among these tasks. Overall, MTL effectively enhanced the generalization ability and accuracy of the 2M-hBCINet model.

#### 5.2.3 Multiband comparison experiments

To further validate the superiority of the 2M-hBCINet model across different EEG frequency bands, we categorized EEG signals into four frequency bands: theta (four to eight Hz), alpha (8–13 Hz), beta (13–30 Hz), and gamma (30–50 Hz), and conducted comparative experiments for each band. The comparison models included the single EEG model ${VAE}_{EEG}-CBA$ , single EMG model ${VAE}_{EMG}-CBA$ , and reference EEG models Reference-Leeb (Leeb et al., 2011), Reference-Barachant (Barachant et al., 2012), and Reference-Hutchison (Hutchison et al., 2010).

**FIGURE 11:**
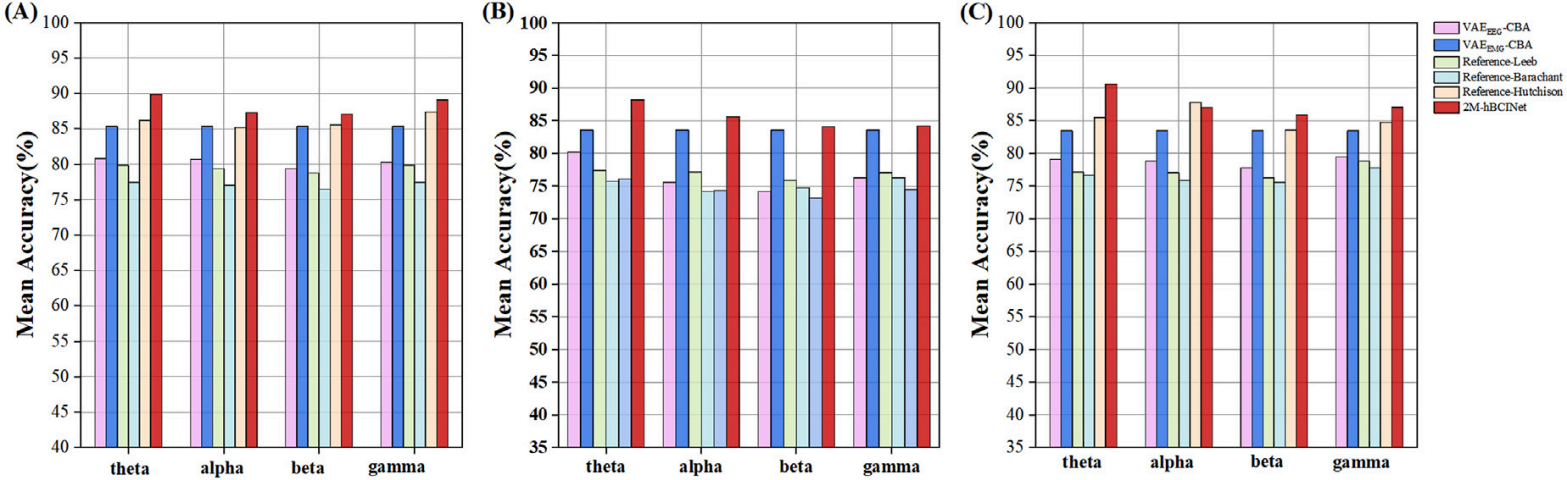
Statistical results of multi-band comparison experiments. The signals were divided into four frequency bands: theta (four to eight Hz), alpha (8–13 Hz), beta (13–30 Hz), and gamma (30–50 Hz), and comparative experiments were conducted for each band. **(A)** In the MI-EEMG dataset, a total of 420 experiments were conducted (6 models × 5 bands × 14 participants). Compared to other models, the 2M-hBCINet model’s average accuracy increased by 4.5%–13.6% for theta, 1.9%–12.9% for alpha, 1.7%–11.2% for beta, and 3.7%–12.8% for gamma bands. **(B)** In the WAY-EEG-GAL dataset, a total of 420 experiments were conducted (6 models × 5 bands × 14 participants). Compared to other models, the 2M-hBCINet model’s average accuracy improved by 4.6%–12.1% for theta, 2.0%–11.4% for alpha, 0.5%–10.9% for beta, and 0.6%–9.7% for gamma bands. **(C)** In the ESEMIT dataset, 780 motor imagery classification experiments were conducted for each learning approach (6 models × 5 bands × 26 participants). Compared to other models, the 2M-hBCINet model demonstrated an average accuracy enhancement of 7.1%–14.3% for theta, 3.5%–11.2% for alpha, 2.4%–11.7% for beta, and 3.6%–10.5% for gamma bands.

**FIGURE 12:**
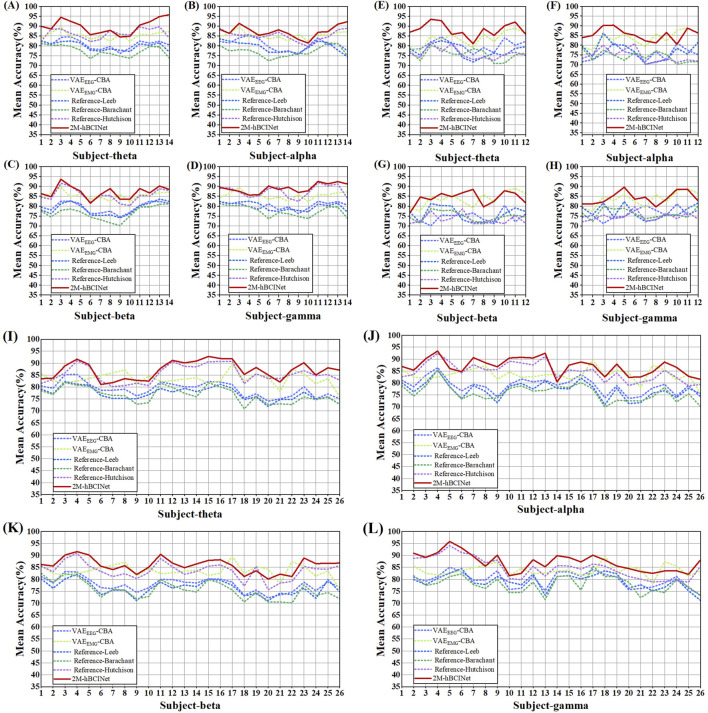
Multi-band comparison experiment performance of each model. **(A)**, **(B)**, **(C)**, and **(D)** describe the performance of each model in the theta (four to eight Hz), alpha (8–13 Hz), beta (13–30 Hz), and gamma (30–50 Hz) bands in the MI-EEMG dataset, respectively. **(E)**, **(F)**, **(G)**, and **(H)** describe the performance of each model in the theta (four to eight Hz), alpha (8–13 Hz), beta (13–30 Hz), and gamma (30–50 Hz) bands in the WAY-EEG-GAL dataset, respectively. **(I)**, **(J)**, **(K)**, and **(L)** describe the performance of each model in the theta (four to eight Hz), alpha (8–13 Hz), beta (13–30 Hz), and gamma (30–50 Hz) bands in the ESEMIT dataset, respectively.

Altogether, 420 experiments were conducted with the MI-EEMG dataset (6 models × 5 frequency bands × 14 participants), and the results are shown in Figure 11A. Figures 12A–D illustrate the performance of each participant across different frequency bands. For the WAY-EEG-GAL dataset, 360 MI classification experiments were performed for each model (6 models × 5 frequency bands × 12 participants), and the results are shown in Figure 11B. Figures 12E–H depict the performance of each participant across different frequency bands in this dataset. A total of 780 MI classification experiments were performed with the ESEMIT dataset for each learning method (6 models × 5 frequency bands × 26 participants), and the results are summarized in Figure 11C. Figures 12I–L illustrate the performance of each participant across different frequency bands.

From Figure 11, it can be observed that in the theta (four to eight Hz), alpha (8–13 Hz), beta (13–30 Hz), and gamma (30–50 Hz) frequency bands, the 2M-hBCINet model achieved average accuracy improvements of 4.5%–13.6%, 1.9%–12.9%, 1.7%–11.2%, and 3.7%–12.8%, respectively, compared to the models ${VAE}_{EEG}-CBA$ , ${VAE}_{EMG}-CBA$ , Reference-Leeb, Reference-Barachant, and Reference-Hutchison for the MI-EEMG dataset. Considering the WAY-EEG-GAL dataset, the improvements were 4.6%–12.1%, 2.0%–11.4%, 0.5%–10.9%, and 0.6%–9.7%. For the ESEMIT dataset, the model demonstrated accuracy increases of 7.1%–14.3%, 3.5%–11.2%, 2.4%–11.7%, and 3.6%–10.5%. Overall, the 2M-hBCINet model exhibited the best performance across all datasets, confirming its superiority. The reason for the optimal performance of the 2M-hBCINet model across different frequency bands is its ability to extract more discriminative EEG features using unsupervised methods. In addition, unlike other models that rely solely on EEG, such as Reference-Leeb, Reference-Barachant, and Reference-Hutchison, the 2M-hBCINet model effectively integrates deep EMG features through the CAM, further enhancing its performance across different frequency bands. This resulted in superior MI classification capabilities across all EEG frequency bands.

#### 5.2.4 Muscle fatigue experiment

**FIGURE 13:**
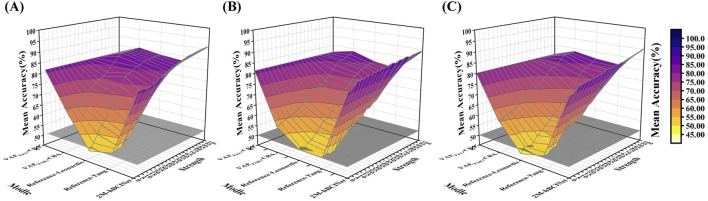
Muscle fatigue test. **(A)**, **(B)**, and **(C)** show the results of visualization comparison of the 2M-hBCINet model with other models. These results are based on 525 experiments (5 models × 21 muscle force states × 5 trials) across the MI-EEMG, WAY-EEG-GAL, and ESEMIT datasets.

In practical MI classification tasks, issues such as muscle fatigue and decreased muscle strength can occur in participants. In this study, a muscle fatigue experiment was designed to evaluate the performance of the 2M-hBCINet model under muscle fatigue conditions. Referencing a previously described method (Xi et al., 2021), we simulated the muscle strength state of participants experiencing fatigue by reducing the amplitude of the EMG signals (with an amplitude reduction resolution of 5%). The comparative models included a single EEG model ( ${VAE}_{EEG}-CBA$ ), a single EMG model ( ${VAE}_{EMG}-CBA$ ), and EMG classification models that used the same feature selection but different classifiers, namely, Reference-Leonardis (Leonardis et al., 2015) and Reference-Tang (Tang et al., 2014). A total of 525 experiments were conducted with the MI-EEMG, WAY-EEG-GAL, and ESEMIT datasets (5 models × 21 muscle strength states × 5 repetitions), and the visualization results are presented in Figure 13.

Figure 13 shows the trends in accuracy for each model across the MI-EEMG, WAY-EEG-GAL, and ESEMIT datasets. For models relying solely on EMG, such as ${VAE}_{EMG}-CBA$ , reference-Leonardis, and reference-Tang, the accuracy dropped to 71.6%–75.7%, 64.6%–77.7%, and 61.2%–69.9% when the muscle strength was 50%. At a muscle strength of 25%, the accuracy further declined to 62.3%–67.8%, 54.8%–60.2%, and 54.5%–57.9%. As the muscle strength continued to decrease, the accuracy of these EMG-only models stabilized at approximately 50%. In contrast, for the proposed EEG-EMG classification model, 2M-hBCINet, the accuracy was 86.1%–87.6% at a muscle strength of 50% and 82.6%–85.6% at 25%. When the muscle strength was further reduced, the accuracy of 2M-hBCINet stabilized at 80.1%–81.3%.

## 6 Conclusion

In this study, EEG and EMG data were collected from the participants, and corresponding datasets were created. A new model for MI classification based on 2M-hBCINet utilizing EEG and EMG signals was proposed. This model first processed the EEG and EMG signals using VAE networks to extract deep feature information specific to each signal. The features were then combined, and different weights assigned to the extracted features using CAM to achieve selective feature optimization. Additionally, the model employed MTL, training simultaneously on the primary task of MI classification and auxiliary tasks of EEG and EMG reconstruction, and a feature metric learning task. Different loss functions were used for each task to enhance the learning effectiveness. Finally, the superiority of the proposed model and its broad applicability under different frequency bands and muscle conditions were validated through ablation, MTL comparison, multi-frequency band comparison, and muscle fatigue experiments based on the LOOCV method. The necessity and effectiveness of each module and training task were verified through ablation experiments.

Despite the model’s excellent performance in deep feature extraction and classification of EEG and EMG signals, only basic preprocessing was performed for the EEG signals. Currently, multivariate iterative filtering (MIF) techniques have shown significant results in EEG signal processing, thereby improving the signal handling accuracy and efficiency. Future work could consider integrating MIF techniques with this model to further enhance its performance.

This approach has broad application potential as an end-to-end MI classification model. Beyond applications in rehabilitation robotics, it can be extended to other fields related to EEG and EMG signals, such as driver anomaly detection, motion-sensing games, and emotion analysis. For driver anomaly detection, the model can monitor driver fatigue, distraction, and emotional states through EEG and EMG signal analyses, thereby enhancing road safety. In the realm of motion-sensing games, the model can provide more intuitive control methods, enriching player experience, while in emotion analysis applications, it can accurately identify individual emotional responses. With continuous technological advancements, potential application scenarios are expected to increase.

## Data Availability

The datasets presented in this article are not readily available because the data collection conditions of the population are harsh. Requests to access the datasets should be directed to Zhenxi Zhao, 15121175203@163.com.
